# Increased risk of deep vein thrombosis in end-stage renal disease patients

**DOI:** 10.1186/s12882-018-0989-z

**Published:** 2018-08-16

**Authors:** Hsueh-Yi Lu, Kuang-Ming Liao

**Affiliations:** 1 Department of Industrial Engineering and Management, National, Yunlin University of Science and Technology, Yun-Lin, Taiwan; 2 Department of Internal Medicine, Chi Mei Medical Center, Chiali, Taiwan

**Keywords:** Deep vein thrombosis, End-stage renal disease, Incidence

## Abstract

**Background:**

Previous studies have shown that chronic kidney disease increases the risk of deep vein thrombosis (DVT). DVT is the risk of pulmonary embolism among persons with end-stage renal disease (ESRD). Information on the incidence of DVT in ESRD is limited, and no studies have been conducted in the Asian population. The aim of our study was to investigate the incidence of DVT in Asian ESRD patients by comparing with the non-ESRD patients and to identify the associated risk factors.

**Methods:**

This study retrieved patients who were diagnosed with ESRD (ICD-9-CM codes 585 or 586) between January 1, 2004, and December 31, 2010, from the National Health Insurance Research Database in Taiwan. All ESRD patients had received a catastrophic illness card from the Ministry of Health and Welfare in Taiwan, with the major illness identified as ESRD. Patients who had DVT before the index date or who had incomplete records were excluded from the analysis. A total of 4865 ESRD patients were enrolled. There are 3564 ESRD patients included after exclusion of patients with previous DVT and patients with incomplete records. The control subjects were randomly selected as the patients without ESRD by matching study subjects according to age (±3 years), gender, and the year of admission at a 2:1 ratio from the same dataset.

**Results:**

The incidence rate of DVT was substantially higher in the ESRD group than in the without-ESRD group (20.9 vs. 1.46 per 10^4 person-years). The adjusted hazard ratio (aHR 13.92; 95% CI 9.25–20.95) of DVT for the ESRD patients was 13.92 times that for the non-ESRD patients. ESRD patients older than 50 years had a higher risk of DVT (aHR 1.65; 95% CI 1.13–2.40; *P* = 0.01). Hyperlipidemia was significantly associated with an increased risk of DVT (aHR 1.73; 95% CI 1.08–2.78; *P* = 0.02). ESRD patients with three or more comorbidities were substantially more likely to have DVT (aHR 1.45; 95% CI 1.03–2.03; *P* = 0.03).

**Conclusions:**

ESRD patients had a higher risk of DVT than non-ESRD patients. Among the ESRD patients, being older than 50 years and having dyslipidemia increased the risk of DVT.

## Background

The epidemiology of deep vein thrombosis (DVT) has been studied in the general population [1–3]. .End-stage renal disease is associated with a 2.3-fold increased risk of DVT compared with the general population [4]. The rate of DVT occurring 1.5 to 3 years after transplantation was 2.9 episodes/1000 person-years. Patients with severe renal function impairment at the end of the first year after renal transplantation increased risk for DVT [5]. Otherwise, there is high risk of DVT recurrence in patients developing a first episode of DVT after renal transplant [6]. The incidence of DVT ranges from 48 to 182 per 100,000 observation-years and increases with age. The risk factors for DVT include old age, surgery, cancer, hospitalization, stasis, immobilization, obesity, trauma, exogenous hormones, pregnancy and inherited thrombophilia [7]. Chronic kidney disease (CKD) is associated with increased procoagulants, including cystatin C, C-reactive protein, interleukin-6, tumor necrosis factor-α soluble receptor 1, intercellular adhesion molecule-1, fibrinogen, and factor VIII [8]. Previous studies have shown that CKD increases the risk of DVT, and its epidemiology and clinical implications have been reported [4, 9–14]. The prevalence of pulmonary thromboembolism has been reported at autopsy [15]. DVT is considered an uncommon disease in patients with end-stage renal disease (ESRD) [16]. A prospective study conducted in a university-affiliated intensive care unit found that ESRD was a risk factor for DVT (hazard ratio (HR) 3.7, 95% confidence interval (CI) 1.2–11.1). To our knowledge, studies examining DVT and ESRD have been limited. The aim of our study was to assess the incidence of DVT in Asian ESRD patients by comparing with the non-ESRD patients and to identify the associated risk factors for DVT within ESRD patients.

## Methods

### Ethics statement

This study was approved by the Institutional Review Board (IRB) of the Chi Mei Medical Center, Taiwan (10609-E02). The anonymity of the selected subjects was preserved by encrypting the original identification information before further analysis. Informed consent was waived by the approving IRB.

### Data sources

The National Health Insurance (NHI) is a public single-payer insurance program that has provided nationwide coverage in Taiwan since 1995. It covers approximately 99% of the population and contracts with 97% of healthcare providers. The NHI Research Database (NHIRD), which is authorized and released for research purposes, contains all the inpatient and outpatient registration and claims data of the NHI program. The data include patients’ demographic characteristics, disease-diagnostic and surgery-operation codes (based on the International Classification of Diseases, Ninth Revision, Clinical Modification [ICD-9-CM]), prescription records, and medical expenditures. The NHIRD is one of the largest administrative health care databases in the world. This study used a longitudinally linked NHIRD dataset, which consists of a cohort of one million randomly selected enrollees traced retrospectively from 1996 to 2010. No statistically significant differences were found in the age, sex, and health care cost distributions of the selected subjects.

### Patients

**Fig. 1:**
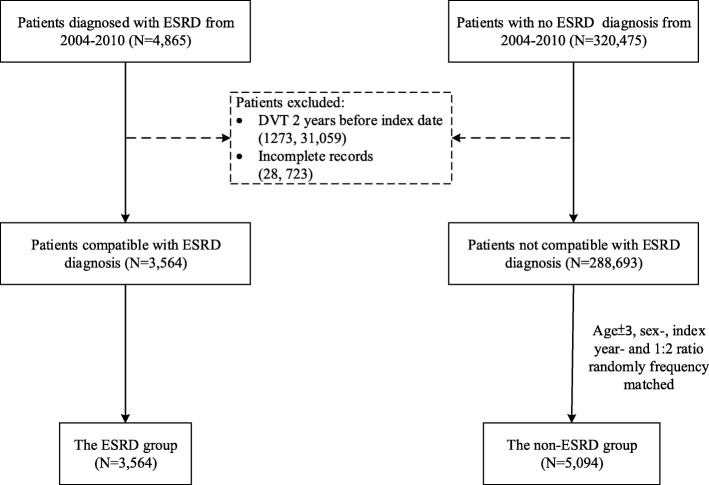
Flowchart of subject enrollment

This study utilized data from patients who were diagnosed with ESRD (ICD-9-CM codes 585 or 586) between January 1, 2004, and December 31, 2010. In Taiwan, patients with ESRD requiring dialysis can apply for a catastrophic illness card and free of any charge. The aim of our study was to assess the incidence of DVT in Asian ESRD patients. Before patients had received a catastrophic illness card of ESRD from the Ministry of Health and Welfare in Taiwan, patients may include the ICD-9-CM codes 585 and 586. We linked the Catastrophic Illness Patient Database file to get the diagnosis of ESRD. Because each individual registered in the catastrophic illnesses database is exempted from any co-payment for treatment, the process for evaluating applicants’ eligibility for this registry is strict and comprehensive. For the case of ESRD, the catastrophic illness certification was issued by a nephrologist and confirmed by another nephrologist. Under this condition, the possibility of ESRD identified in our study being true positive is considerably high. The application date of the catastrophic illness card was designated the index date as a starting time point to estimate the risk of DVT. Patients who had DVT before the index date or who had incomplete records were excluded from the analysis. A total of 4865 qualified patients who had ESRD were preliminarily identified before the exclusion filtering (Fig. 1).

### Outcomes and comorbidities

The control subjects were randomly selected as the patients without ESRD by matching study subjects according to age (±3 years), gender, and the year of admission at a 2:1 ratio from the same dataset. To investigate the risk of DVT, the patients started from the index date were followed until DVT (ICD-9-CM code 453.8), death, or withdrawal from the NHI occurred or the end of 2010. Comorbidities, including hypertension, diabetes, hyperlipidemia, cerebrovascular accident, congestive heart failure, atrial fibrillation, lower leg fracture or surgery, and cancer, were identified from 1 year before the index date according to ICD-9-CM codes from the outpatient and inpatient records.

### Statistical analysis

Demographic and comorbidity variables were expressed as frequencies (percentages) or means (±standard deviations, SDs). The continuous variables were distributed normally after normality test. The demographic characteristics were gender and age (stratification in the following age groups: ≤39, 40–49, 50–59, 60–69, and over 70 years). The incident rates of DVT were estimated using the total number of events divided by the total follow-up period (per 10,000 person-years).

Cox proportional hazards models were conducted to compare the risk of DVT between ESRD and non-ESRD groups. Joint effects with associated risk factors (age and comorbidities) for the risk of DVT in the ESRD group were also analyzed by Cox proportional hazards models. The covariate variables included gender and comorbidities. Proportionality of hazards assumption was tested and fulfilled before cox regression was conducted. Hazard ratios (HRs) and their 95% confidence intervals (CIs) were estimated by Cox regression. Cumulative incidence functions of DVT for different groups were plotted and compared using competing risk methods [17]. All statistical tests were two-sided, and a *P*-value of 0.05 was considered significant.

## Results

### Patient characteristics

The ESRD group comprised 3564 patients. The mean age was 62.76 ± 14.69, 35.7% of patients were aged ≥70 years, and 49.4% of patients were male (Table 1). Similarly, the non-ESRD group had 5094 patients. The mean age was 61.84 ± 15.00 years, 33.1% of patients were aged ≥70 years, and 49.1% were male. The distributions of age and gender among both groups were similar. Patients in the ESRD group had a significantly higher prevalence of comorbidities than the non-ESRD group in atrial fibrillation (1.8% vs. 0.5%), hypertension (56.8% vs. 26.9%), diabetes (39.4% vs. 13.4%), hyperlipidemia (5.8% vs. 1.2%), cerebrovascular accident (11.1% vs 2.7%), congestive heart failure (17.3% vs 1.3%), lower leg fracture or surgery (1.2% vs 0.5%), and cancer (2.3% vs 1.9%).

**Table 1:** Demographic characteristics and comorbidities in patients with and without ESRD

	Non-ESRD (*n* = 5094)	ESRD (*n* = 3564)
Age		
≦39	412 (8.1^a^)	265 (7.4)
40–49	677 (13.3)	406 (11.4)
50–59	1137 (22.3)	772 (21.7)
60–69	1180 (23.2)	849 (23.8)
≧70	1688 (33.1)	1272 (35.7)
Mean ± SD	61.84 ± 15.00	62.76 ± 14.69
Gender		
Male	2499 (49.1)	1762 (49.4)
Female	2595 (50.9)	1802 (50.6)
Deep vein thrombosis	26 (0.5)	200 (5.6)
Comorbidities		
Atrial fibrillation	24 (0.5)	64 (1.8)
Hypertension	1369 (26.9)	2024 (56.8)
Diabetes	684 (13.4)	1404 (39.4)
Hyperlipidemia	63 (1.2)	208 (5.8)
Cerebrovascular accident	135 (2.7)	394 (11.1)
Congestive heart failure	65 (1.3)	616 (17.3)
Lower leg fracture or surgery	28 (0.5)	44 (1.2)
Cancer	99 (1.9)	82 (2.3)

Abbreviations: *ESRD* end-stage renal disease, *SD* standard deviation

^a^Percentage

### Incidence of DVT

In Table 2, the incident rate of DVT was substantially higher in the ESRD group than in the group without ESRD (20.9 vs. 1.46 per 10^4 person-years). The adjusted HR (aHR 13.92; 95% CI 9.25–20.95) of DVT indicated that the risk of DVT for the ESRD patients was 13.92 times that of the non-ESRD patients. This relationship was further investigated to examine the associations among age, gender, and comorbidities. In the ESRD group, the DVT incidence rates stratified by age (≤30, 40–49, 50–59, 60–69 and over 70 years) had corresponding aHRs that increased accordingly (12.94, 13.54, 20.01, 25.55, and 25.30). Similarly, incidence rates that increased with age were found in the non-ESRD group. The Cox regression models showed that age, gender, and comorbidities were associated with higher risks of DVT in the ESRD groups than in the non-ESRD group (Table 2). For example, for the patients with hypertension, the adjusted HR of DVT in the ESRD group was 13.21 (95% CI 6.46–27.02; *P* < 0.001) times that of the non-ESRD group.

**Table 2:** Incidence of DVT in patients with and without ESRD

Characteristics	Non-ESRD (n = 5094)			ESRD (n = 3564)				
	Event	TFP (PY)	IR	Event	TFP (PY)	IR	aHR (95% CI)	*P*
DVT	26	18,112.37	1.46	200	9552.35	20.94	13.92 (9.25–20.95)	< 0.001
Age								
≦39	2	1767.13	1.13	13	1004.83	12.94	10.99 (2.48–48.73)	0.002
40–49	1	2729.76	0.37	20	1477.39	13.54	36.76 (4.93–273.0)	< 0.001
50–59	5	4334.96	1.15	47	2348.80	20.01	15.96 (6.34–40.15)	< 0.001
60–69	7	4303.83	1.63	58	2270.50	25.55	15.25 (6.95–33.46)	< 0.01
≧70	11	4976.70	2.21	62	2450.81	25.30	11.06 (5.81–21.06)	< 0.01
Gender								
Male	10	8833.28	1.13	91	4745.28	19.18	16.31 (8.49–31.36)	< 0.001
Female	16	9279.10	1.72	109	4807.06	22.67	12.46 (7.37–21.08)	< 0.01
Comorbidities								
Hypertension								
Yes	8	4974.73	1.61	125	5714.18	21.88	13.21 (6.46–27.02)	< 0.001
No	18	13,137.64	1.37	75	3838.16	19.54	13.61 (8.13–22.79)	< 0.001
Diabetes								
Yes	4	2515.32	1.59	89	3607.59	24.67	15.01 (5.50–40.93)	< 0.01
No	22	15,597.06	1.41	111	5944.75	18.67	12.72 (8.05–20.10)	< 0.01
Hyperlipidemia								
Yes	0	217.13	0	19	525.90	36.13	34.41 (0.50–2985)	.102
No	26	17,895.24	1.45	181	9026.45	20.05	13.17 (8.73–19.88)	< 0.01
Cerebrovascular accident								
Yes	1	391.38	2.55	18	901.44	19.97	7.48 (1.00–56.07)	0.05
No	25	17,720.00	1.41	182	8650.90	21.04	14.29 (9.40–21.71)	< 0.01
Congestive heart failure								
Yes	0	184.50	0	30	1304.25	23.00	24.50 (0.20–3057)	.194
No	26	17,927.88	1.45	170	8248.09	20.61	13.68 (9.05–20.68)	< 0.01
Lower leg fracture or surgery								
Yes	0	90.47	0	4	87.07	45.94	68.62 (0.02–241,330)	.310
No	26	18,021.90	1.44	196	9465.28	20.70	13.71 (9.10–20.65)	< 0.01
Cancer								
Yes	1	281.68	3.55	3	153.36	19.56	4.40 (0.46–42.45)	.201
No	25	17,830.70	1.40	197	9398.99	20.96	14.30 (9.43–21.69)	< 0.01

Abbreviations: *ESRD* end-stage renal disease, *DVT* deep vein thrombosis, *TFP* total follow-up period, *PY* per 10,000 person-years, *IR* incident rate per 10,000 person-years, *aHR* adjusted hazard ratio

### DVT occurring with other risk factors

The joint effects for DVT between ESRD and associated risk factors were investigated further. As shown in Table 3, the ESRD patients older than 50 years had a higher risk of DVT (aHR 1.65; 95% CI 1.13–2.40; *P* = 0.01) indicated that the risk of DVT for the ESRD patients over 50 years old was 1.65 times that of the younger ones. The association between comorbidities and DVT for ESRD patients was further investigated. Hyperlipidemia was significantly associated with an increased risk of DVT (aHR 1.73; 95% CI 1.08–2.78; *P* = 0.02). ESRD patients with three or more comorbidities had a substantially higher risk of DVT (aHR 1.45; 95% CI 1.03–2.03; *P* = 0.03).

**Table 3:** Joint Effects for DVT between ESRD and Associated Risk Factors (n = 3564, IR = 5.6%)

Variable	Number (%)	Events	Rate	aHR (95% CI)	*P*
Age > =50 years	2893 (81.20)	167	0.06	1.65 (1.13–2.40)	0.01
Hypertension	2024 (56.80)	125	0.06	1.10 (0.83–1.47)	0.52
Diabetes	1404 (39.39)	89	0.06	1.26 (0.95–1.67)	0.11
Hyperlipidemia	208 (5.84)	19	0.09	1.73 (1.08–2.78)	0.02
Cerebrovascular accident	394 (11.05)	18	0.05	0.90 (0.56–1.47)	0.68
Congestive heart failure	616 (17.28)	30	0.05	1.04 (0.70–1.53)	0.86
Atrial fibrillation	64 (1.80)	2	0.03	0.77 (0.19–3.10)	0.71
Lower leg fracture or surgery	44 (1.23)	4	0.09	2.04 (0.76–5.48)	0.16
Cancer	82 (2.30)	3	0.04	0.89 (0.28–2.78)	0.84
≥1 comorbidity	2547 (71.46)	151	0.06	1.14 (0.83–1.58)	0.43
≥2 comorbidities	1509 (42.34)	88	0.06	1.13 (0.86–1.50)	0.39
≥3 comorbidities	630 (17.68)	43	0.07	1.45 (1.03–2.03)	0.03

Abbreviations: *ESRD* end-stage renal disease, *DVT* deep vein thrombosis

### Cumulative incidence functions of DVT

**Fig. 2:**
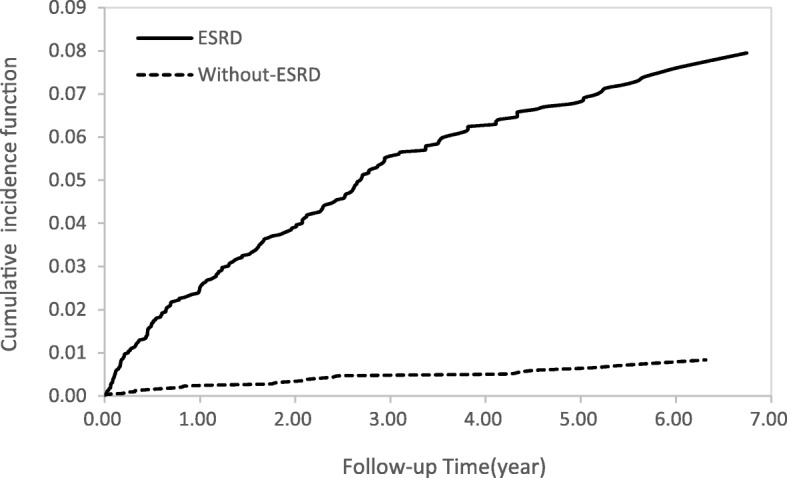
Cumulative incidence function of DVT in patients with and without ESRD

**Fig. 3:**
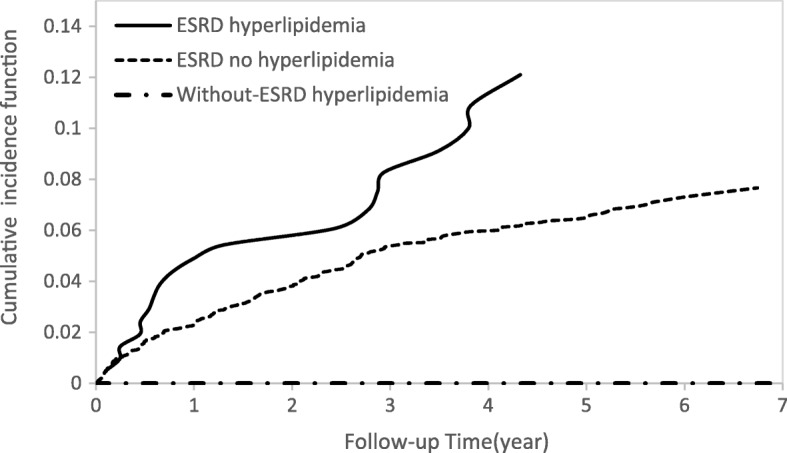
Cumulative incidence function of DVT in patients with hyperlipidemia

**Fig. 4:**
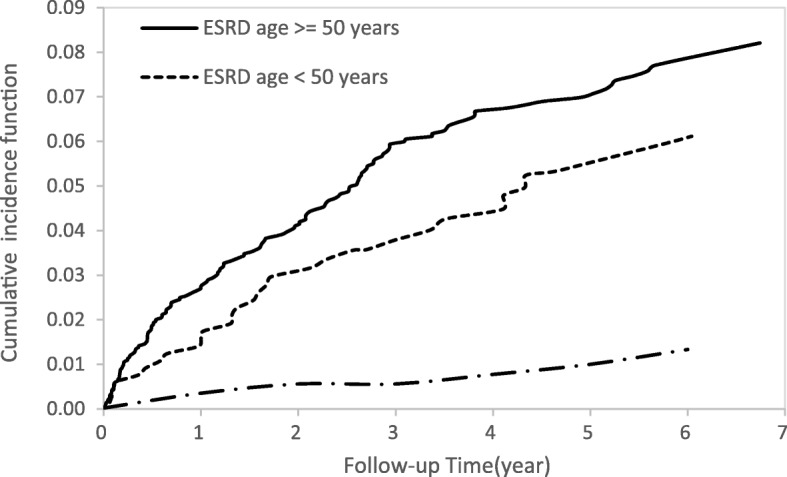
Cumulative incidence function of DVT for patients aged over 50 years

**Fig. 5:**
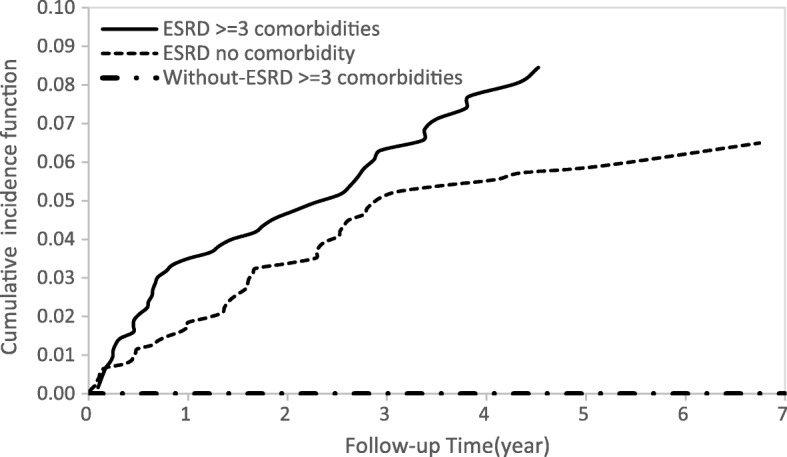
Cumulative incidence function of DVT for patients with three or more comorbidities

Cumulative incidence functions for the ESRD and non-ESRD groups illustrating the occurrence of DVT over time (Fig. 2) showed differences between the two study groups. In Fig. 3, ESRD patients with hyperlipidemia had a higher risk of DVT than ESRD patients with no hyperlipidemia and patients without ESRD with hyperlipidemia. Similar patterns of higher risk of DVT were also found for ESRD patients older than 50 years (Fig. 4) and for patients with 3 or more comorbidities (Fig. 5).

## Discussion

Previous studies examining DVT in patients with ESRD were case reports or case series or reported adverse outcomes in clinical trials [16, 18–21], and some of these patients with DVT had risk factors for DVT such as malignancy, postoperative state, or prolonged hospitalization. There is little evidence and no previous epidemiological studies showing that ESRD itself predisposes individuals to DVT. To our knowledge, this is the first study that used a national medical database to evaluate the epidemiology of DVT in patients with ESRD. In our study, the incidence of DVT was 20.94 per 10,000 person-years, and ESRD patients had a higher risk of DVT than patients without ESRD, with an aHR of 13.92 (95% CI 9.25–20.95) in an Asian population. The incidence of DVT and pulmonary embolism was lower in Asian country compared with Western ones. Previous study showed ESRD is associated with a 2.3-fold increased risk of DVT compared with the general population^4^. In Taiwan, ESRD patients was about 14-fold increased risk of DVT compared with non-ESRD patients. Treatment dyslipidemia and increased awareness in patients aged 50 or more are important for management DVT in Asian ESRD populations.

### ESRD and DVT

The coagulation cascade is a complicated process that involves platelets, endothelial cells and coagulation factors. A bleeding tendency was frequently observed in clinical practice in ESRD patients, but thrombotic events, such as arteriovenous fistula thrombosis, peripheral arterial occlusive disease and DVT, were also noted in ESRD. According to the previous review data, the factors contributing to thrombotic events include hemodialyzer-induced platelet aggregation; elevated plasminogen activator inhibitor-1; increased Von Willebrand factor release; oxidative stress; increased homocysteine; fibrinogen; activity of factors VII, VIII, and IX–XII; thromboplastin (tissue factor); fibrinopeptide A; and reduced protein C [22]. In addition, there is a possibility that the clinical and demographic characteristics of the ESRD population continued to change, and treating anemia with erythropoietin or blood transfusion was associated with DVT [22].

According to previous literature and our previous study, DVT is less likely to occur among Asians than among Caucasians [23]. For example, the incidence of DVT in an Asian COPD population was 18.78 per 10,000 person-years, with an aHR of 1.38 (95% CI 1.06–1.80) compared to a non-COPD population, after adjusting for age, sex, atrial fibrillation, hypertension, diabetes, hyperlipidemia, cerebrovascular accident, congestive heart failure, lower leg fracture and cancer, and the incidence was lower than that in the Caucasians population. This study showed that the incidence of DVT was higher in the ESRD population than in the non-ESRD population in Asians. To our knowledge, there is no recently published data regarding the incidence of DVT in ESRD patients. According to previous studies utilizing administrative data in 2002, the incidence of venous thromboembolism, including DVT and pulmonary embolism, was 149.9 events per 100,000 dialysis patients [24]. The lower incidence rate may be related to data errors or incorrect coding [4].

### Age and DVT

Age is a risk factor for DVT. According to previous studies, DVT was rare in young individuals, and the risk of DVT increased with age, or approximately 1% per year in the elderly [25]. In our study, we found that the incidence of DVT increased with age in the ESRD population. Compared to patients without ESRD, the highest incidence of DVT in the ESRD population occurred between 40 and 49 years of age, with an aHR of 36.76. ESRD placed patients aged between 40 and 49 years at greater risk for developing DVT. In addition, the aHR of DVT in the ESRD population was 1.65 for adults older than 50 years compared with adults younger than 50 years.

### Comorbidities and DVT

Compared to patients without ESRD, patients with ESRD had some comorbidities that increased the risk of DVT, such as hypertension and diabetes. The well-known risk factors for the development of DVT, such as lower leg fracture or surgery and cancer, were not statistically significant for DVT because the number of cases of DVT in the non-ESRD population was zero or only one case. Further, the incidence of DVT was much lower in Asian populations than in Western populations. Future epidemiological studies on the incidence of DVT in patients with ESRD and their risk factors in Western populations may be needed.

In the ESRD population, being over 50 years old and having dyslipidemia were the only two risk factors for DVT in our study. Low incidence of DVT and a small case number for some comorbidities, such as atrial fibrillation, lower leg fracture or surgery, and cancer, may hamper these risk factors from reaching statistical significance. In our study, ESRD patients with more than three risk factors had a significantly increased risk of DVT.

### ESRD and acute kidney injury in DVT

A population-based study enrolled 19,073 cases and authors found that CKD increased the risk of DVT [7]. A study in Taiwan that also used NHIRD [26] found that acute kidney injury increased the risk of DVT compared to that in patients with normal renal function. We not only studied the incidence of DVT in the ESRD population but also investigated the risk factors for DVT between ESRD and non-ESRD patients. In addition, we also surveyed the risk factors for DVT in the ESRD population. Future studies can clarify the association between DVT and renal function by delineating the risk of DVT at different stages compared to that of the normal population.

### Limitations

The first of our limitations was the small number of cases of ESRD patients with comorbidities of lower leg fractures or surgery and cancer, which totaled only 87 and 153 person-years, respectively. Lower leg fracture or surgery and cancer are well-documented risk factors for DVT, but there is no significant correlation statistical significance in our study owing to a small number of cases. Second, one may doubt the accuracy of the database. In the NHI in Taiwan, patients with ESRD who required hemodialysis can apply for a catastrophic illness certificate and do not need to pay a co-payment for outpatient or inpatient care. This application will be formally reviewed by other physicians. The risk of incorrect coding is very low, and the validation of the NHIRD had been performed [27, 28]. Otherwise, previous study from Canadian claim database has showed the diagnosis of DVT and pulmonary embolism in adult was sensitive by using ICD-9-CM [29]. Third, there are inherent limitations to this database. There is no smoking history, no body mass index data, no laboratory data and no image data. Otherwise, the potential information bias due to misclassification of DVT should be considered. In our study, we investigated the incidence of DVT in ESRD patients and their risk factors from a large nationwide database based on physician diagnosis in clinical practice. The comprehensive enrollment can represent real-world populations.

## Conclusions

The incidence of DVT was higher in ESRD patients than in the non-ESRD population. Being older than 50 years and having dyslipidemia are risk factors for DVT among ESRD patients in Asian populations.
